# Emerging role of mesenchymal stem cell-derived extracellular vesicles in oral and craniomaxillofacial tissue regenerative medicine

**DOI:** 10.3389/fbioe.2022.1054370

**Published:** 2022-11-29

**Authors:** Meng Liu, Xin Liu, Yuting Su, Shijie Li, Yuan Chen, Anqi Liu, Jing Guo, Kun Xuan, Xinyu Qiu

**Affiliations:** ^1^ State Key Laboratory of Military Stomatology and National Clinical Research Center for Oral Diseases and Shaanxi International Joint Research Center for Oral Diseases, Department of Preventive Dentistry, School of Stomatology, Fourth Military Medical University, Xi’an, Shaanxi, China; ^2^ Department of Orthodontics, School of Stomatology, Fourth Military Medical University, Xi’an, Shaanxi, China; ^3^ Center of Clinical Aerospace Medicine, School of Aerospace Medicine, Fourth Military Medical University, Xi’an, Shaanxi, China

**Keywords:** mesenchymal stem cells, extracellular vesicles, regenerative medicine, tissue engineering, oral and craniomaxillofacial tissue

## Abstract

Mesenchymal stem cells (MSCs) are multipotent stem cells with differentiation potential and paracrine properties, drawing significant attention in the field of regenerative medicine. Extracellular vesicles (EVs), mainly including exosomes, microvesicles and apoptotic bodies (ABs), are predominantly endosomal in origin and contain bioactive molecules, such as miRNAs, mRNAs, and proteins, which are transferred from their original cells to target cells. Recently it has emerged that MSC-derived EVs (MSC-EVs) combine the advantages of MSCs and EVs, which may be used as a promising MSC-based therapy in tissue repair and regeneration. Oral and craniomaxillofacial diseases are clinically complications containing the soft and hard tissues in craniofacial and dental arches. These diseases are often induced by various factors, such as chemical, microbiological, physical factors, and systemic disorders. For decades, tissue repair and regeneration in oral and craniomaxillofacial regions provide substantial improvements in the prevention and treatment of some severe diseases. In this review we discuss MSC-EVs and their therapeutic potential in oral and craniomaxillofacial tissue regenerative medicine.

## 1 Introduction

Oral and craniomaxillofacial regions consist of soft and hard tissues like muscles, bone, cartilage and skin, which brings great obstacles for therapies in oral and craniomaxillofacial diseases, including infectious disease such as caries and periodontitis, function disorders like temporomandibular joint (TMJ) disorders, and maxillofacial tissue defects caused by tumors, trauma and deformities (Ding et al., 2020). To deal with different diseases, multiple treatments have been developed, among which the requirements for tissue reconstruction arises because of congenital deformities, trauma through sports and other accidents in oral and craniomaxillofacial regions (Gaihre et al., 2017). However, conventional tissue reconstruction with designed biomaterials encounters different limitations such as confined biocompatibility, frequent skin or mucosa irritations. These limitations have brought out urgent demands for new remedies in this field.

Mesenchymal stem cells (MSCs) have been isolated from various tissues, such as bone marrow, adipose tissue, umbilical cord, and dental pulp (Keshtkar et al., 2018). Due to its low immunogenicity, great self-renewal potential and multidirectional differentiation, MSCs have been widely used in the field of tissue engineering and regenerative medicine (Keshtkar et al., 2018; Chen et al., 2020), which exhibited remarkable capacity on promoting tissue repair in oral and craniomaxillofacial diseases. However, it has been observed that exogenous MSCs only survived a short time after transplantation, failing to proliferate and differentiate in quantity, which impacts the therapeutic effects (Hoogduijn and Lombardo, 2019), leading to further investigation and modification of MSCs. In the beginning, researchers found that MSCs interact with recipient cells through differentiation and paracrine signaling pathway, while recent studies reveal that MSCs play a vital role in treatments of various diseases mainly by secreting extracellular vesicles (EVs) as well as soluble paracrine factors (Sigmarsdottir et al., 2020). EVs contain various bioactive molecules for communication and interaction between donor and recipient cells, which maintain life function and normal development of cells.

This finding provides opportunities to develop novel cell-free therapeutic strategies in oral and craniomaxillofacial diseases. At present, MSCs-based therapy still suffers from considerable limitations in the following aspects: i) difficult to determine best culture conditions and proper administration mode, ii) hard to detect and quantitate in real-time due to individual differences after MSCs transplantation, iii) adverse reactions to MSCs injection, such as venous thrombosis (Furlani et al., 2009), iv) low survival rate of MSCs after transplantation (Warrier et al., 2022). Inspiringly, since EVs are cell-free, MSC-derived EVs (MSC-EVs) are safer and more stable than MSCs. The application of MSC-EVs has covered these disadvantages to some extent. The application of MSC-EVs makes it possible for quantitative index and quality control in tissue regenerative medicine. Therefore, research of therapy based on MSC-EVs has attracted more and more attention in the field of oral and craniomaxillofacial tissue regenerative medicine.

## 2 Extracellular vesicles

### 2.1 Characteristics and isolation of EVs

**FIGURE 1:**
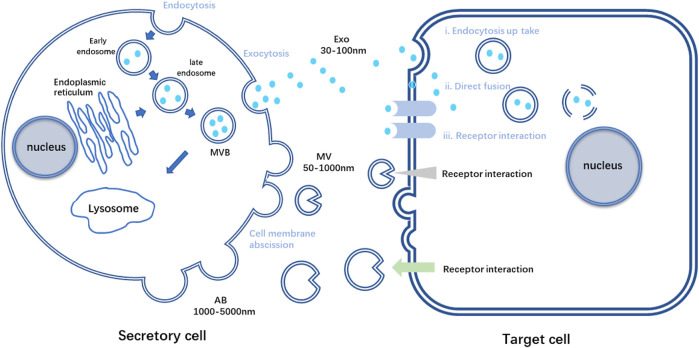
Schematic diagram of the process of secretory cells secreting three kinds of EVs and the process of target cells receiving three kinds of vesicles. AB, apoptotic bodies; Exo, exosome; MV, Microvesicles; MVB, multivesicular bodies.

EVs are nano-vesicles with lipid bilayers secreted by almost all cell types. Based on the vital role MSCs played in tissue repair and its strong connection with EVs *via* paracrine signaling pathways, MSC-EVs have gradually entered the scene in tissue engineering and regenerative medicine (Keshtkar et al., 2018). Researchers have found that various peptides and glycoproteins exist on the membrane of EVs, which is crucial for intercellular recognition and adhesion. EVs are relatively stable in circulation, and these distinct membrane landmarks can influence their tropism to specific organs. It is reported that different integrins on membrane enable EVs to accumulate in brain, liver or lungs, depending on the particular integrin type (Hoshino et al., 2015). What’s more, bioactive molecules EVs contained during their formation are in plenty, such as lipids, proteins, and nucleic acids (van Niel et al., 2018). Once released into the extracellular space, EVs can reach recipient cells and deliver their contents to elicit the corresponding functional responses (Figure 1). Through these molecules, also called EV cargoes, EVs participate in intercellular communication and interaction frequently, acting like loaded vehicles, shuttling between cells to realize molecular information transferring (Panda et al., 2021). However, the mode of vesicle interaction with the cell surface and the mechanisms that mediate the transfer of EV cargoes are not completely unraveled until nowadays. These procedures are complex and relate to the origins of EVs and the recipient cells, linking to the downstream effects and processes (van Niel et al., 2018).

Current studies focus on utilizing EVs as candidates for cell-free therapy or targeted drug delivery system due to its ability of loading biological macromolecules (Sun et al., 2021), while the role of EVs played in the regenerative medicine is still under investigation. Compared with exogenous MSCs transplantation in regenerative medicine, MSC-EVs show greater advantages because of its lower immunoreactivity and biological stability. Since MSC-EVs have no potential to differentiate, they are also more controllable and possess higher biosecurity. The studies have reported that MSCs were rapidly removed within 48 h after administration, which restricted its direct effects on tissue repair (Liu et al., 2020). On the contrary, EVs are characterized with extended half-life and excellent penetration in drug delivery (Kunter et al., 2007; Lai et al., 2013). The cell-free conditioned medium of MSCs demonstrates similar therapeutic effects on tissue repair (Gnecchi et al., 2005). Therefore, EVs based cell-free therapy has become an emerging hot spot and may gradually replace MSC-based therapy.

In view of the promising prospect of MSC-EVs in regenerative medicine, it is important to isolate and apply EVs in tissue repair. There are various methods to isolate EVs, which demonstrate different advantages and disadvantages in terms of purity, time, and cost (Crescitelli et al., 2021). Among all these methods, ultracentrifugation is one of the most common and convenient. Firstly, cells and debris are deposited and removed by centrifugation. Then the supernatant containing biological macromolecules undergoes further ultra-centrifugation to concentrate EVs in the pellet. Specifically, to isolate ABs, inducing cell apoptosis is necessary before centrifugation (Liu et al., 2018). Even though it is easy to implement, this method costs a long time. In addition, aggregation of different types of EVs during ultracentrifugation largely affect the purity, it is impossible to specifically separate each type of EVs only by centrifugation. As for therapy or experiment, EVs need to be isolated and concentrated while MSCs are strictly removed. Hence, many studies have utilized size-exclusion chromatography to isolate EVs, through which particles not in the required size range are excluded. However, passing through the filters by force may lead to the deformation and rupture of large vesicles and platelets, resulting in a loss of purity. Moreover, it costs lots of time to pass through multiple filters with different sizes (György et al., 2011). Due to the immunoaffinity of specific proteins on surfaces of EVs, immunoaffinity separation have been utilized (Clayton et al., 2001). Nevertheless, for each specific type of EVs, the specific antibody should be selected, which leads to low universality and inefficiency (Théry et al., 2006). There are several methods available for combined application, including polymerization precipitation, microfluidics, and so on (Chen et al., 2010; Yamada et al., 2012; Witwer et al., 2013). After isolation, identification of the acquired “EVs” is necessary. The common methods include observing morphological characteristics of “EVs” by electron microscopy, measuring particle size distribution by dynamic light scattering (DLS) analysis, and detecting molecular markers by western blot or immunofluorescence.

### 2.2 Main classes of EVs

Since there is no consensus emerged on specific markers of EVs subtypes so far, some measurable and operational terms are used to classify EVs. For example, size was referred to define EV subtype as “small EV” (sEVs, < 100 nm or < 200 nm) and “medium/large EVs” (m/lEVs, > 200 nm). Biochemical composition, condition description or origin of cells were also recommended to distinguish different EVs (Théry et al., 2018). However, it is still wide accepted that EVs are divided into the following three categories according to biogenesis, particle size and surface markers (Table 1).

**TABLE 1:** Comparison of basic physiological characteristics of three kinds of EVs.

Characteristics	Exosome	Microvesicles	Apoptotic bodies
General characteristics	Almost all cells can secrete		
	Presented in body fluids		
	Rich in protein, lipid and genetic material		
Biogenesis mechanism	Endocytosis and exocytosis, Generated by multi vesicle system	Cell membrane budding	Release during apoptosis
Diameter	30‐150 nm	50‐1000 nm	1000‐5000 nm
Main markers	Transmembrane protein (CD9, CD63, CD81)TSG101, Lactadherin, LAMP1	CD40PhosphatidylserineCell surface antigen	Phosphatidylserine

#### 2.2.1 Exosomes

Exosomes were first discovered and named when researchers were investigating reticulocytes transformation (Johnstone et al., 1987). As the smallest kind of EVs, exosomes are formed by exocytosis, ranging in diameters from 30 nm to 150 nm. Exosomes, also referred as intraluminal vesicles (ILVs), originate from early endosomes and are produced by inward budding of the endosomal membrane. With the participation of Golgi apparatus, ILVs mature into multivesicular bodies (MVBs), which either undergo metabolization and excretion in lysosomes, or fuse to plasma membrane and eventually release its contents, including exosomes, to the extracellular space (Pant et al., 2012). The biogenesis of exosomes requires the participation of both complex proteins and enzymes, which constitute endosomal sorting complexes required for transport (ESCRT) pathway. With the development of electron microscope, the structure of exosomes has been observed, which are constrained by lipid bilayers, with different markers on the membrane. CD9, CD63, and CD81are discovered to be located on the outer membrane and they are the most common surface markers for exosomes sorting, both of which contain four transmembrane domains (Jansen et al., 2009; Kosaka et al., 2010; Salunkhe et al., 2020). Previous studies have found that major histocompatibility complexes I and II (MHC-I and MHC-II) exist on the membrane of exosomes, indicating the important role exosomes playing in regulating immune responses, like presenting antigens, activating macrophages and T cells with recognition and elimination (Wolfers et al., 2001; Blanchard et al., 2002; Segura et al., 2005). This feature of exosomes has two sides, it boosted the interest in further exploration of immune dysregulation in tumors by modifying the tumor immunity of exosomes while compromised their therapeutic use in transplantation with potential risk of immunological rejection. However, exosomes derived from MSCs can avoid immune rejection comfortingly, which can compensate for exosomes. Besides, exosomes are reported to be enriched in glycoproteins, proteins related to phosphorylation and so on (Doyle and Wang, 2019). As a result, to improve the sorting accuracy, it is necessary to explore more sensitive and specific molecular markers for composite screening.

Existing in various body fluids and secretions, exosomes reflect the physiological and pathological state of human body. For example, exosomes isolated from alveolar lavage fluid of asthmatic patients contain unique miRNAs, which can be used as the prospective indicators for asthmatic (Levänen et al., 2013). In terms of treatment, it has been found that MSC-derived exosomes reduced monocytes infiltration and thus inhibited inflammation by downregulating MCP-1, an attractant of monocytes (Yu et al., 2016). Despite this, the important role of exosomes in regenerative medicine has been explored. Recent studies confirmed that MSC-derived exosomes enhance the wound healing of skin burn injury by delivering several proteins (Wu et al., 2018). Some studies also verified that noncoding RNAs in exosomes play a crucial role in bone regeneration (Yin et al., 2021). What’s more, the possible future potential for microRNA-containing exosomes to treat peripheral nerve injuries are also recognized (Qing et al., 2018).

#### 2.2.2 Microvesicles

Microvesicles (MVs) are formed by the shedding of plasma membrane, ranging in diameters from 150 nm to 1,000 nm. MVs were initially considered as secretions of cells for removing wastes, while the procoagulant property of MVs has been found in the subsequent studies (Loyer et al., 2014; Desrochers et al., 2016; Xie et al., 2019). MVs are formed by the budding of and pinching of the plasma membrane, which is the result of the dynamic interaction between phospholipid redistribution and cytoskeletal protein contraction. Actin myosin is involved in the budding process, so it depends on intracellular calcium ions. The membrane of MVs is rich in lipids such as cholesterol, sphingomyelin and ceramide, and the specific surface marker is like CD40 (Phelps et al., 2018). It is reported that MVs are responsible for the transport of genetic regulatory molecules like mRNAs and miRNAs, which are effective by the interaction of specific receptors and ligands after contacting with the target cells (Keshtkar et al., 2018). Meanwhile, MVs protect the genetic regulatory molecules from degradation by RNA enzymes in the blood.

Recently, the significant roles of MSC-derived MVs (MSC-MVs) in anti-inflammatory regulation and tissue repair have been demonstrated. It has been reported that MSC-MVs improved repair upon lung injury *via* transferring mRNA for angiopoietin1 (Ang1) to endothelial cells, which increased the expression of connexin (Hu et al., 2018). The study has shown that MVs act as intercellular messengers in cutaneous wound healing (Laberge et al., 2018), which has brilliant future in tissue repair. With further investigation, the researchers discovered that the therapeutic effect of transplanted cells may be associated with their ability to release MVs. These may because that MVs take part in intercellular communication, transferring bioactive molecules like DNAs, mRNAs and miRNAs. Because of their nanoscale size and unique subcellular structures, MVs can be applied as physiological mediators in tissue regeneration (Agrahari et al., 2019).

#### 2.2.3 Apoptotic bodies

As the largest type of EVs, apoptotic bodies (ABs) are released by apoptotic cells, ranging in diameters from 1,000 nm to 5,000 nm, and expressing cell death markers like Caspase-3 and Annexin V (Akers et al., 2013). In fact, apoptotic cells also release vesicles with the similar particle size of exosomes and MVs, which are described as the apoptotic microvesicles (Akers et al., 2013; Poon et al., 2014; Atkin-Smith et al., 2015). As a programmed cell death manner, apoptosis plays an important role in tissue development, cell renewal and homeostasis maintenance, during which molecular signals are released to induce tissue regeneration. Studies have shown that ABs are involved in the regulation of bone homeostasis, metabolic homeostasis, and vascularization during tissue regeneration (Liu et al., 2018; Liu et al., 2020; Zheng et al., 2021). Meanwhile, like their parental cells, ABs are responsible for wastes disposure. Thus, it is reasonable to speculate that there is signal transmission between ABs and macrophages, the professional phagocytes closely related to inflammation and metabolism (Zheng et al., 2021).

Macrophages are the major cell type responsible for clearing apoptotic cells by efferocytosis, through which inhibiting inflammatory responses (Perry et al., 2019; Boada-Romero et al., 2020). It has been found that cadherin anchored on the surface of ABs, mediating the uptake of ABs by macrophages, and reducing the infiltration and activation of macrophages in the sick liver (Zheng et al., 2021). To our knowledge, M2 macrophages play an important role in suppressing inflammation and promoting tissue repair (Smith et al., 2017). It has been observed that proteins able to induce macrophage polarization to M2 phenotype existed on the surface of ABs, which made the ABs beneficial to bone homeostasis and metabolic homeostasis. Moreover, in type II diabetic (T2D) mice, it has been demonstrated that MSC-derived ABs are targeted to macrophages and played vital roles in immunological regulation of macrophages, with remarkable improvements in lowering the chronic inflammation induced by diabetes (Castegna et al., 2020; Zheng et al., 2021). Therefore, there is a close relationship between ABs and macrophages, which may lead to future application of ABs in tissue repair and regenerative medicine. As a kind of underestimated EVs, the functions of ABs need to be further explored.

## 3 Functions of EVs

### 3.1 Intercellular communication

Intercellular interactions mainly rely on the molecular signal transmission and modification of target genes, which regulates biological functions of cells (Zhang et al., 2021). Through direct contact and fusion with the plasma membrane, molecular signals are transferred to the target cells by endocytosis of EVs. For example, exosomes have been found in the intracellular space of the marginal region of the heart membrane in myocardial ischemia mice, which has been proved to be linked with intercellular interaction (Sahoo and Losordo, 2014). Except for regulating inflammation and oxidative stress to protect cells from hypoxia and ischemia-reperfusion, the therapeutic effects of exosomes depend on angiogenesis to resist tissue ischemia, which requires communication among endothelial cells, stromal cells, and stem cells. Moreover, it has been discovered that endothelial cell-derived exosomes stimulate endothelial cell migration and angiogenesis through the miR-214 dependent pathway (van Balkom et al., 2013). The EVs isolated from the conditioned medium cultured with CD34^+^ stem cells promote angiogenesis both *in vivo* and *in vitro* (Sahoo et al., 2011). It has also been reported that CD34^+^ stem cells transported Shh protein into other cells partially through exosomes with beneficial effects, while injecting Shh protein directly into the heart showed no therapeutic effects. Thus, the protective effects of exosomes on Shh protein degradation by protease have been indirectly proved, which prolonged the half-life of Shh (Mackie et al., 2012). Similarly, EVs isolated from the blood of patients with glioblastoma contain specific mRNAs, which not only shows the protective effects, but also indicates EVs as biomarkers for diseases like glioblastoma (Skog et al., 2008). All the evidence demonstrated that EVs play a crucial role in intercellular communication, accelerating signal transmission between cells.

### 3.2 Immunomodulation

A remarkable feature of MSCs is immunomodulation. Besides inherited genes, MSCs are affected by surrounding cytokines and receptors on plasma membrane, such as toll like receptors (TLRs) (Costa et al., 2021). Thereby, MSCs tend to exhibit more than one phenotype. Many studies have shown that TLR4 and LPS activated the MSC1 phenotype expressing pro-inflammatory mediators, while interferon-y (INF-y) and tumor necrosis factor α (TNF-α) activated TLR3 and promoted the differentiation towards MSC2 phenotype, which regulated peripheral immune cells (Naftali-Shani et al., 2017; Ferreira et al., 2018; Almeria et al., 2019; Ananthakrishnan et al., 2020). However, the functions of MSCs cannot be controlled after transplantation, and the secretion of immunomodulating factors from MSCs cannot be activated by chronic or minor inflammation (Gonzalez-Pujana et al., 2020). As a kind of secretion, the cargos of EVs are determined by the parental cells (Liang et al., 2019). Therefore, isolating EVs from cultured MSCs has attracted more attention. After culturing MSCs in specific environment and inducing MSCs to express corresponding phenotype, MSC-derived EVs then extracted may be a more controllable and safer therapeutic tool. The recent study has shown that TNF-α pretreated MSCs produced exosomes containing miR-146a, which inhibited the activation of fibroblasts and inflammatory responses in the model of urethral fibrosis (Liang et al., 2019). Additionally, EVs derived from IL-1β-pretreated MSCs contained more miR-146a, which were transferred into macrophages to induce M2 polarization and thus inhibit inflammation (Song et al., 2017).

### 3.3 Targeted delivery system

It is acknowledged that paracrine factors, like vascular endothelial growth factors (VEGF), transforming growth factors (TGF), interleukin-1 receptor antagonists (IL-1RA), are imperative in mediating biological functions of MSCs, including immunomodulation, and promoting tissue repair (Christodoulou et al., 2018). However, the soluble factors have no ability to target cells and exist only a short time, which hinders the clinical transformation. Transportation through EVs not only protect soluble factors from degradation, but also position them to target cells through the receptor-ligand system on the membranes (Li et al., 2017). Thus, EVs have become a promising candidate for targeted drug delivery. Once used for drug delivery, the requirements on safety, yield and targeting must be met. It can be considered to increase the number of MSCs and stimulate the secretion ability of MSCs to expand the production of EVs. Although MSCs possess strong ability of self-renewal, the unlimited proliferation is unrealistic. It is far from enough to depend on ligand-receptor system for positioning the target cells, as a result, modifying surface markers of EVs has been considered. Certainly, appropriate targeted peptides and approaches should be selected according to the specific target region (Tian et al., 2018). At present, the common modification methods include genetic engineering, covalent modification, and non-covalent modification (Salunkhe et al., 2020). For example, to improve the distribution level of exosomes in brain and weaken the metabolism, targeting effect on glioma have been achieved by binding nerve targeting peptides to the outer membrane of exosomes (Jia et al., 2018). According to recent studies, the distribution of iron oxide nanoparticles (IPON) after intravenous injection can be controlled by external magnetic field. Thus, IPON has been loaded into MSC-EVs, and the modified EVs have been found guided to the target area by the external magnetic field. Moreover, it can also be used to detect the distribution of EVs both qualitatively and quantitatively (Kim et al., 2020).

## 4 MSC-EVs in tissue repair and regeneration in oral and craniomaxillofacial regions

**FIGURE 2:**
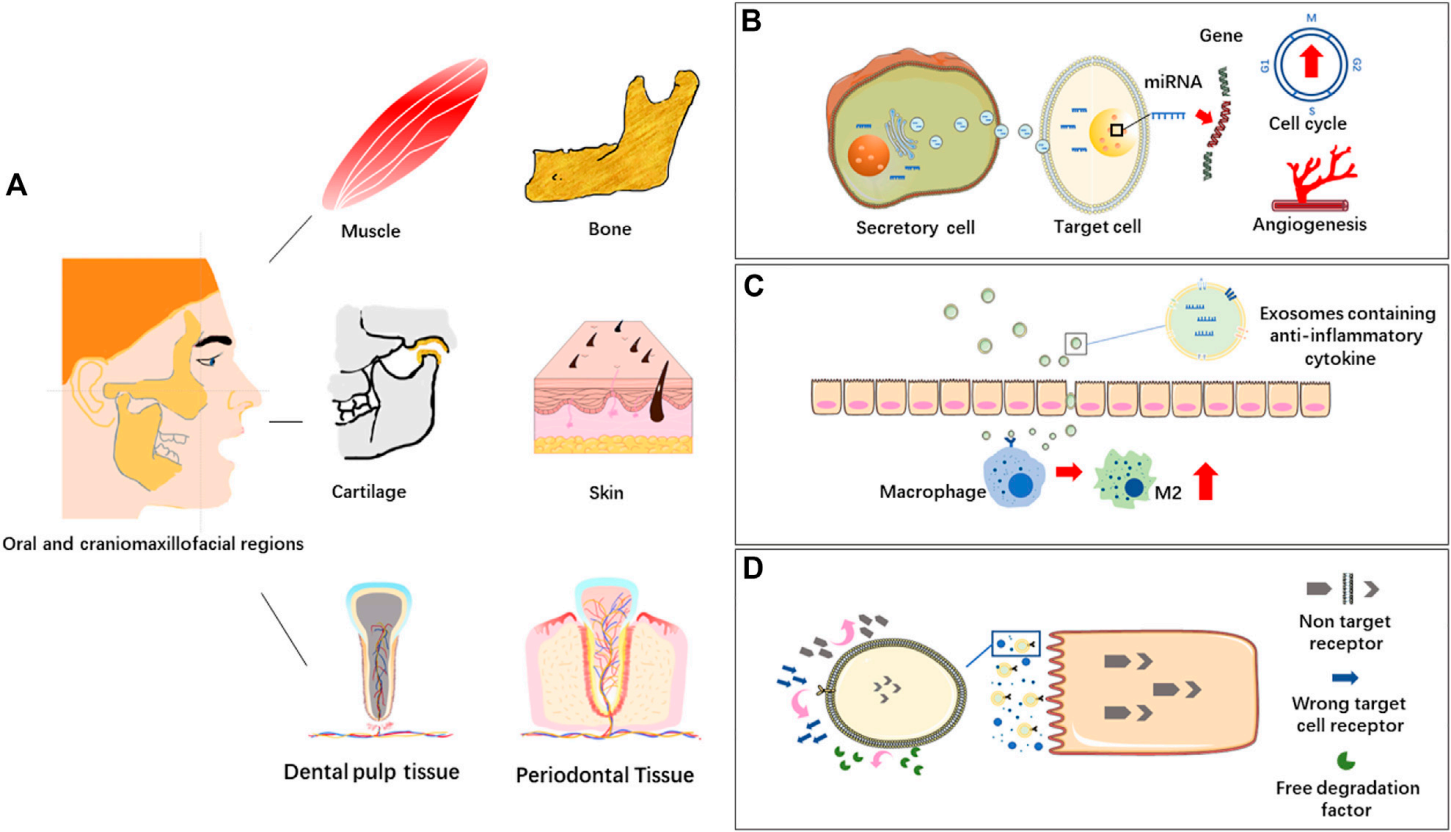
**(A)** Oral and craniomaxillofacial tissues. **(B)** EVs in intercellular communication. Extracellular vesicles (EVs) transport bioactive molecules like miRNAs to regulate gene expression of target cells, affecting cell life activities, such as angiogenesis and cell cycle. **(C)** EVs in immunomodulation. EVs containing immunomodulating factors are transferred into macrophages to induce M2 polarization and thus inhibit inflammation. **(D)** EVs in targeted delivery system. EVs bind to target cells through the receptor-ligand system on the membranes.

Different soft and hard tissues co-exist in oral and craniomaxillofacial regions, such as muscle, bone, cartilage, skin and dental pulp tissue. MSC-EVs derived from different types of mesenchymal stem cells can response or react through different biomolecules or signaling pathways in tissue repair and regeneration, which is a new research hotspot in regenerative medicine (Figure 2).

### 4.1 Muscle

Normal healing process of muscle injuries depends on the differentiation of endogenous stem cells, while excessive disordered growth often leads to scar formation, due to inflammation and other factors (Sharma and Maffulli, 2006). It is the endogenous stem cells which largely determine the self-healing capability, the functional signalling pathways and regulatory molecules vary from tissue to tissue. In young xenopus laevis model, the role of Ca^2+^ signalling pathway and extracellular signal regulated kinase 1/2 (ERK1/2) in regeneration of spinal cord and muscle have been investigated. It has been proved that ERK1/2 was activated in stem cells of the damaged area. In skeletal muscle associated tissues, the activation of ERK1/2 was necessary for an injury-induced increase in intracellular store-dependent Ca^2+^ dynamics, but in spinal cord, injury increases Ca^2+^ influx-dependent Ca^2+^ activity independent of ERK1/2 signalling (Levin and Borodinsky, 2022). It is obvious to see the vital role of stem cells in muscle repair, and the purpose is to regulate the inflammatory environment by stimulating transformation of stem cells. Thus, the infiltration of inflammatory cells is reduced, and extracellular matrix (ECM) is deposited orderly (Sousa-Victor et al., 2022). Additionally, in a muscle injury mouse model, local application of MSC-EVs at the wound site reduced the formation of fibrotic tissue and promoted angiogenesis, and then improved muscle regeneration (Nakamura et al., 2015). Pretreating MSCs with specific conditions may induce the secretion of EVs with specific functions. In the acute myocardial infarction model, MSC cultured under hypoxia for 72 h significantly released EVs which promoted blood flow recovery, reduced the infarct area, and improved cardiac function (Bian et al., 2014). Therefore, stem cells and stem cell secretions are required for muscle regeneration, which stimulating the proliferation and differentiation of the remaining stem cells, reducing the impact of inflammation on the damaged environment.

### 4.2 Bone

The bone tissue in craniomaxillofacial region is mostly irregular with rich blood supply, which not only means the strong anti-infection ability, but also indicates more chances of injury-caused severe inflammation. The bone density varies slightly according to the site, and the craniomaxillofacial bone tissue contains more pores, manifesting a high possibility of fracture. Moreover, bone defect induced by tumors and infections severely impact the function and aesthetics of the craniomaxillofacial region, thus the repair and regeneration of craniomaxillofacial bone tissue has always been a complicated clinical problem. The difficulty is that the immune responses and immunological rejection result in the rejection of implanted biomaterials, no matter autogenous bone tissue or biosynthetic materials. Meanwhile, hypoxia, lack of cells and blood vessels in the damaged area led to nonhealing wound and even bone necrosis (Marx, 1983). Hence, angiogenesis is of great importance for bone regeneration. It has been pointed that MSC-EVs activated various signaling pathways in endothelial cells to stimulate sprouting and capillaries formation *via* transferring abundant contents. Besides, MSC-EVs transported Wnt3 protein into endothelial cells to enhance angiogenesis (McBride et al., 2017). Immunomodulation is one of the important functions of EVs, which making differences in solving the inflammation in radiation-induced osteonecrosis (ORN) (Pu et al., 2020). The major goal in immunomodulation is to transform macrophage phenotype from pro-inflammatory M1 to anti-inflammatory M2 by regulating the microenvironment, such as miR-146 and miR-34 (Jiang et al., 2012; Domenis et al., 2018). Wnt/β-catenin signaling pathway is closely involved in macrophage polarization towards M2 phenotype. EVs loaded with activate Wnt/β-catenin signaling pathway and alleviate radiation induced bone loss (Zuo et al., 2019). In addition, EVs transport metal-lothionein-2, which promote the anti-inflammatory effects of macrophages, and participate in NO mediated osteogenesis (Li et al., 2020). Dental pulp stem cells (DPSCs) also play an important role in the treatment of bone defects, it has been reported that co-culture of DPSCs and ADSCs significantly enhanced the osteogenic ability, compared to culture alone. DPSC-derived EVs promote osteogenic differentiation of ADSCs by enhancing the phosphorylation of ERK1/2 and JNK, and thus activating MAPK signaling pathway. The similar co-culture systems are effective approaches, less expensive than adding specific growth factors (Jin et al., 2020). Currently, there are various kinds of seed cells, the combining application of which show excellent therapeutic potential. EVs derived from MSCs which pretreated by TNF-α can change the composition of miRNAs, thereby controlling macrophage phenotype to control inflammation balance, create a good environment, and promote osteogenesis process (Kang et al., 2022). Meanwhile, it has been found that MSC-EVs promoted osteoblast proliferation and differentiation through miR-122-5p and MAPK signaling pathway (Zhao et al., 2018).

### 4.3 Cartilage

Unlike limbs, bone tissue in craniomaxillofacial region is mostly hard bone with limited mobility. It is only TMJ that moves in craniomaxillofacial region, in which functions of articular cartilage play important roles. Articular cartilage is the elastic tissue covered the end of the joint with no blood supply nor nerves. It is surrounded by ECM rich in collagen and proteoglycan, which supports and buffers the joint (Carballo et al., 2017). Therefore, regulation on surrounding environment of articular cartilage is key to its repair and regeneration. Based on the outstanding performance of MSCs in tissue engineering, many studies have shown that MSCs from synovial joint tissue had better cartilage forming ability than that from non-joint tissues (Huang et al., 2017). Moreover, with science and technology advanced, the focus of studies gradually shifted to EV-based therapy (Ruiz et al., 2016). It has been reported that EVs derived from MSCs not only promoted cartilage regeneration, but also downregulated TNF-α mediated COX-2 and pro-inflammatory interleukins, inhibiting collagenase activity. Adding MSC-EVs to chondrocytes isolated from osteoarthritis patients also promoted the secretion of proteoglycan and cartilage regeneration (Vonk et al., 2018). Moreover, these secretions were encapsulated in EVs, and these EVs fused with target cells to release the contents (Yin et al., 2016; Tao et al., 2017a). A recent study showed that activated platelet-rich plasma (PRP) upregulated platelet-derived growth factor AB (PDGF-AB), transforming growth factor-β (TGF-β) and VEGF, which were secreted through EVs. These factors were secreted in PRP-derived EVs (PRP-EVs) to promote cell proliferation (with reduced apoptosis) and cartilaginous matrix secretion *via* suppressing the Wnt/β-catenin signal pathway in interleukin-1β (IL-1β)-stimulated chondrocytes, which were harvested from the terminal of tibia and femur of rabbits (Liu et al., 2019). The study has shown that injection of infrapatellar fat pad derived EVs (IPFP-EVs) inhibited apoptosis, enhanced matrix synthesis, and reduced the production of catabolic cytokines like MMP-13 (Wu et al., 2019). In addition to the bioactive molecules secreted under physiological condition, it is also effective to modify stem cells to obtain desired abilities. Wnt5a and Wnt5b carried by exosomes activate yes associated protein (YAP) through Wnt signaling pathway, which enhances the proliferation and migration of chondrocytes while significantly reduces the secretion of ECM. However, the high expression of miR-140-5p block it through RalA. Therefore, gene modification of EVs avoid the side effects, and enhance the proliferation and migration of articular chondrocytes (Tao et al., 2017b). Thus, modifying cells to acquire specific EVs can be used as a potential treatment, beneficial to tissue repair and regeneration in cartilage.

### 4.4 Skin

Skin injuries are common disease caused by trauma and resection of large tumor. However, long healing time often leads to excessive scar formation, which bring burden to patients psychologically and physiologically (Frykberg and Banks, 2015). With further research on MSCs, it has been widely used in cutaneous wound healing, which opened a new situation for tissue engineering and regenerative medicine (Marofi et al., 2019). It has been found that MSCs transported hepatocyte growth factor (HGF) through EVs and promoted the proliferation of a variety of cells (Yang et al., 2021). HGF stimulates the activation of MMP-2 and MMP-9, participating in angiogenesis, cell migration and fiber remodeling, and supporting cutaneous wound healing with reduced scar formation (Yoshida et al., 2004). It has been found that EVs secreted by induced pluripotent stem cells (iPSCs) derived MSCs (iMSCs) activated ERK-1/2 signal pathway and promoted the growth of human keratinocytes and human dermal fibroblasts. Moreover, epidermal growth factor (EGF) and epidermal growth factor receptor (EGFR) are very important for the stability of the environment in the epidermis and hair follicles. Therefore, it can be inferred that EVs may induce ERK1/2 by activating EGFR-Ras-Raf signaling pathway, to stimulate skin growth (Okamoto, 2010; Kim et al., 2018). In recent years, the important role of EVs in wound healing has become more and more obvious. Exosomes derived from MSCs stimulated re-epithelialization, increased expression of cytokeratin 19 (CK19), promoted synthesis of type I collagen and improved cutaneous regeneration. In the rat burn model, EVs induced Akt signaling pathway and reduced apoptosis caused by heat stress (Zhang et al., 2015). Umbilical cord mesenchymal stem cells (UCMSC) derived EVs promoted transformation of dermal fibroblasts into myofibroblasts through TGF-β1/Smad2/3 signaling pathway and improved the cutaneous wound healing by promoting epithelialization and angiogenesis (Hu et al., 2020; Zhao et al., 2020). Even in systemic sclerosis (SSC), a rare disease characterized by the development of skin fibrosis, MSC-EVs slow down the disease process and make differences (Rozier et al., 2021). In general, EVs are widely used in cutaneous wound healing and the beneficial effects have been showed both in preclinical and clinical research.

### 4.5 Dental pulp tissue

Dental pulp is neurovascular tissue surrounded by hard tissues, which is prone to inflammation. The common risk factors are caries, trauma, and retrograde infection of periodontitis, which lead to serious infection and necrosis on dental pulp and periapical tissues (Mu et al., 2020). To cure diseases in dental pulp tissue, drug therapy and root canal therapy are used as common treatments. However, after the pulp is damaged or extracted, the teeth cannot get nutrition, increasing the fragility and decreasing the resistance of hard tissues, resulting in elevated risk of fracture. Therefore, it is of great significance to protect and restore dental pulp tissue. The researchers attempted to regenerate dental pulp tissue by introducing blood into the root canal from the tissue around the root tip, promoting tissue growth with instruments (Ostby, 1961; Nygaard-Ostby and Hjortdal, 1971). The potential of DPSC-derived EVs in inducing odontogenic differentiation has been explored. It has been found that the combination of exosomes and matrix proteins leaded to the adhesion of biomaterials and endocytosis of DPSCs, to triggering p38/MAPK signaling pathway and promote odontogenic differentiation and tissue regeneration (Huang et al., 2016). DPSC-derived EVs isolated from both normal conditions and odontogenic conditions have been detected to confirm the miRNA sequences. The results showed that EVs isolated from odontogenic conditioned medium up-regulated DSP, dmp-1, ALP, and Runx2 proteins to induce odontogenic differentiation of DPSCs. At the same time, miRNAs in exosomes down-regulated recessive TGF-β binding protein 1 (LTBP1), promoting odontogenic differentiation mediated by TGF-β1/Smad signaling pathway (Hu et al., 2019). Moreover, deciduous autologous tooth stem cells have been implanted into necrotic immature permanent anterior teeth to produce pulp dentin complex, including functional dental pulp tissue regeneration with vasculature, innervation, and the lining odontoblast layer. Furthermore, the regenerated pulp tissues functionally promoted root elongation and apical hole closure. Thus, the physiological function of regenerated dental pulp has been proved, which is of great significance in promoting the development of immature permanent teeth (Xuan et al., 2018). Subsequently, mechanisms under-lying the bioengineering teeth constructed by polymer combined with acellular dental matrix in whole tooth regeneration have been explored. Increased EVs produced by the co-culture of human exfoliated deciduous teeth (SHED) aggregate and acellular dental matrix were found, indicating that the microenvironment provided by acellular dental matrix promoted the odontogenic differentiation. It is an important step in the treatment to accomplish teeth regeneration after complete dislocation (Guo et al., 2021). Therefore, regeneration of dental pulp tissue is continually moving forward.

### 4.6 Periodontal tissue

Teeth are surrounded by periodontal tissue, which participates in supporting and stabilizing the teeth. Once the periodontal tissue is destroyed, alveolar bone resorption and loss of periodontal attachment will happen. Periodontitis is a kind of infectious disease with microorganism as the main pathogenic factor. Prevention is most important for treating periodontitis, accompanied with treatment for infection. If periodontitis develops to the late stage, it should be treated with certain regenerative therapy (Zijnge et al., 2012). Periodontal ligament stem cells (PDLSC) derived EVs contain miR-155-5p, which regulates the balance between Th17 and Tregs by targeting sirtuin-1 in periodontitis (Zheng et al., 2019). The phenotypic transformation of macrophages also plays an important role in the regulation of local immune environment. Bacteria activate macrophages to release pro-inflammatory factors, such as IL-1β and TNF-α, inducing inflammatory cells infiltration, destroying soft tissue and leading to alveolar bone resorption (Spiller and Koh, 2017). Destruction of alveolar bone by T cells and neutrophils is stimulated, which leads to the differentiation of osteoclasts in periodontal cells and the gradual loss of alveolar bone by increasing the local expression of Receptor Activator of Nuclear Factor-κ B Ligand (RANKL) (Darveau, 2010; Hienz et al., 2015). Interestingly, periodontitis compromised dental pulp stem cells secrete EVs carrying miR-378 to promote local angiogenesis to activate the Hedgehog/Gli1 signaling pathway (Zhou et al., 2021). Therefore, the stem cells in the damaged area have certain application ability. It has been reported that EVs derived from SHED were injected into the bone defective area in periodontitis model. The results revealed that EVs had therapeutic effects on bone defects to the same extent as the parental stem cells, and specifically promoted the osteogenesis and inhibited adipogenesis (Wei et al., 2020). Meanwhile, human MSC-EVs loaded on collagen sponge was applied in rat models of alveolar bone defect, and the regeneration of alveolar bone and functional periodontal ligament fibers were observed. Further study proved that MSC-EVs promoted the proliferation and migration of periodontal ligament cells and realized the regeneration of defective periodontal tissue by activating Akt/ERK signaling pathways (Chew et al., 2019). In conclusion, MSCs and MSC-EVs are widely used in tissue repair and regeneration in periodontitis (Table 2).

**TABLE 2:** Effects of MSC-EVs in various tissue repair and regeneration relevant to oral and craniomaxillofacial regions.

	Stem cell type of MSC-EVs	Type of EVs	Target tissue	Biomolecules/Signaling pathways	Mechanism of treatment effect	References
1	MSC	EVs	Muscle	Ca^2+^, ERK 1/2	ERK1/2 in muscle tissue is necessary for calcium signaling, but not in the spinal cord	Levin and Borodinsky, (2022)
2	ADSC	EVs	Skin	ADSC-EVs, MMP-9	ADSC-EVs inhibit downregulating MMP-9 and improve collagen deposition	Wang et al. (2022)
3	ADSC	Exos	Nerve	ADSC-Exos	Enhance the expression and differentiation of PC12 cells into neurons	Shariati Najafabadi et al. (2021)
4	BMMSC	Exos	Vessel	Wnt3a	BMMSC-Exos transport Wnt3a exteriorly and enhance dermal fibroblast proliferation, migration, and angiogenesis *in vitro*	McBride et al. (2017)
5	BMMSC	Exos	Bone	Wnt/β-catenin signaling pathway, Exos	BMMSC-Exo loaded with Wnt can activate Wnt/β-Catenin signal transduction and alleviate bone loss caused by radiation	Zuo et al. (2019)
6	BMMSC	EVs	Bone	Cordycepin	Cordycepin promotes osteogenesis of BMMSCs and accelerates fracture healing *via* hypoxia	Li et al. (2020)
7	DPSC ADSC	EVs	Bone	ERK1/2, JNK, MAPK signaling pathway	When DPSC is co cultured with ADSC, the osteogenic ability is significantly enhanced. DPSC-EV can promote the osteogenic differentiation of ADSC through MAPK pathway by enhancing the phosphorylation of ERK1/2 and JNK	Jin et al. (2020)
8	MSC	Exos	Bone	miRNA	MSC-EVs pretreated with TNF-α can change miRNA composition, thereby controlling macrophage phenotype to control inflammatory balance and promote osteogenesis	Kang et al. (2022)
9	MSC	Exos	Bone	MAPK signaling pathway	MSC-Exo could promote the proliferation of hFOB 1.19 through MAPK signaling pathway, thus alleviating the progression of osteoporosis	Zhao et al. (2018)
10	BMMSC	EVs	Cartilage	TNF-α, COX-2, collagenase	BMMSC-EV can down regulate TNF-α mediated COX-2 and pro-inflammatory interleukin levels inhibit collagenase activity and promote cartilage regeneration	Vonk et al. (2018)
11	MSC	Exos	Cartilage	Akt/Bad/Bcl-2 signaling pathway	PRP-Exos have the capability to prevent GC-induced apoptosis in a rat model of ONFH by promoting Bcl-2 expression *via* the Akt/Bad/Bcl-2 signal pathway under ER stress	Tao et al. (2017a)
12	hUCMSC	Exos	Skin	Wnt4	hUCMSC-Exo contributes to skin wound healing by transmitting Wnt4	Zhang et al. (2015)
13	hUCMSC	Exos	Skin	TGF-β1/Smad 2/3 signaling pathway	hUCMSC-Exo suppress dermal fbroblasts-myofbroblats transition *via* inhibiting the TGF-β1/Smad 2/3 signaling pathway	Hu et al. (2020)
14	MSC	EVs	Skin	miR-29a-3p	MSC-EVs alleviate systemic sclerosis *via* miR-29a-3p	Rozier et al. (2021)
15	DPSC	Exos	Tooth	miRNAs, TGF-β1/smads signaling pathway	Lineage specific exosomes can transfer miRNA in TGFbeta1/smads signaling pathway to induce odontogenic differentiation of human dental pulp stem cells	Hu et al. (2019)
16	PDLSC	Exos	Periodontal	miRNA-155-5p, Th17 Treg	In chronic periodontitis, exosomal microRNA-155-5p in PDLSCs can be used to regulate sirtuin-1 to achieve Th17/Treg balance	Zheng et al. (2019)
17	DPSC	EVs	Periodontal	miRNA-378a, Hedgehog/Gli1 signaling pathway	Periodontitis-compromised DPSCs secrete EVs carrying miRNA-378a promote local angiogenesis by targeting Sufu to activate the Hedgehog/Gli1 signaling	Zhou et al. (2021)
18	SHED BMMSC	Exos	Periodontal	Runx2, p-Smad5	SHED-Exo directly promoted BMMSC osteogenesis, differentiation and bone formation	Wei et al. (2020)
19	BMMSC	Exos	Periodontal	Akt/ERK signaling pathway	MSC exosomes can increase PDL cell migration and proliferation through CD73 mediated adenosine receptor activation of survival promoting Akt and ERK signaling	Chew et al. (2019)

Note: Biomolecules/Signaling pathways refers to the biomolecules or signaling pathways through which MSC-EVs, work out in target tissue.

## 5 Conclusions and future perspectives

The application and mechanism of EVs are one of the hot spots of current research. Because EVs can be produced by almost all types of MSCs, MSC-EVs take part in different intercellular communication in different tissues. Based on the immunoregulatory function and regenerative ability, MSC-EVs are widely used as a specific biological macromolecule in the paracrine signalling pathway (Kordelas et al., 2019). Using MSC-EVs not only avoids the difficulty of obtaining and culturing MSCs, but also can quantitatively and qualitatively modify the desired substances to act on directly. The advantages of MSC-EVs therapy are: (a) The source of MSC-EVs is plentiful and the collection method is relatively simple (Ragni et al., 2020), (b) MSC-EVs have good biocompatibility and stability (Papait et al., 2022), (c) The molecular structure of MSC-EVs is small and can pass through the blood-brain barrier (Ophelders et al., 2016), (d) MSC-EVs can avoid some risks associated to MSC-based therapy like venous thrombosis. Therefore, cell-free therapy based on MSC-EVs is quietly emerging.

Oral and craniomaxillofacial regions contain various tissues, such as muscle, bone and cartilage, which are targets for extensive infections and pathological conditions. Complex composition and complicated environment in oral and craniomaxillofacial regions bring out some situations which cannot be cured by drugs and surgeries, for instance, large bone defects and skin injuries. Current therapeutic strategies, such as bone regenerative growth factors and allografts, have inevitable limitations such as lower osteogenic capacity, immunological rejection, and morbidity at the donor site, when tissue repair and regeneration are needed (Dimitriou et al., 2011). Owing to this increasing demand, there is a rapid development in regenerative medicine mediated by MSC-EVs, satisfying the needs of remedies like bone reconstruction in areas of large defects (Lakshmi et al., 2020).

As illustrated above, MSC-EVs can be produced by different types of MSCs, which helps to fulfill complicated tissue repair needs in oral and craniomaxillofacial regions, such as muscle, bone, cartilage, skin, dental pulp tissue and periodontal tissue. Plentiful treatments are developed with participation of MSC-EVs due to their low immunogenicity and multidirectional function. However, until nowadays, the application of EVs is not unlimited. There is no consensus met on measurement standards and industry regulations for the concentration and dose of specific treatment by using EVs (Théry et al., 2018). Meanwhile, the specific working mechanism of different EVs is not clear. Moreover, the safety of the modified EVs cannot be fully guaranteed. With the rapid development of science and technology, it is believed that through continuous exploration, application of MSC-EVs in tissue repair and regenerative medicine will embrace a brighter future, not only in the field of oral and craniomaxillofacial regions.
